# Spent embryo culture medium metabolites are related to the *in vitro* attachment ability of blastocysts

**DOI:** 10.1038/s41598-018-35342-2

**Published:** 2018-11-19

**Authors:** Fiona D’Souza, Shubhashree Uppangala, Gitanjali Asampille, Sujith Raj Salian, Guruprasad Kalthur, Riccardo Talevi, Hanudatta S. Atreya, Satish Kumar Adiga

**Affiliations:** 1 Department of Clinical Embryology, Kasturba Medical College, Manipal Academy of Higher Education, Manipal, India; 2 NMR Research Centre, Indian Institute of Science, Bangalore, India; 3 Solid State and Structural Chemistry Unit, Indian Institute of Science, Bangalore, India; 4 Dipartimento di Biologia, Università di Napoli “Federico II”, Complesso Universitario di Monte S Angelo, Napoli, Italy

## Abstract

The metabolomic profile of an embryo culture medium can aid in the advanced prediction of embryonic developmental potential and genetic integrity. But it is not known if this technology can be used to determine the *in vitro* potential of inner cell mass (ICM) in adherence and proliferation. Here, we investigated the developmental potential of mouse 2-cell embryos carrying cisplatin-induced DNA lesions (IDL), beyond blastocyst stage using ICM outgrowth assay. The genetic integrity of ICM cells was determined by comet assay. The metabolic signatures of spent medium were recorded 84 hours post injection of hCG (hpi-hCG), and after 96 hours of extended *in vitro* culture (Ex 96) by NMR spectroscopy. We observed that blastocysts that lack the ability to adhere *in vitro* had an increased requirement of pyruvate (p < 0.01), lactate (p < 0.01), and were accompanied by a significant reduction of pyruvate-alanine ratio in the culture medium. We propose that the aforementioned metabolites from 84 hpi-hCG spent medium be further explored using appropriate experimental models, to prove their potential as biomarkers in the prediction of implantation ability of *in vitro* derived human embryos in clinical settings.

## Introduction

Assisted reproduction technology (ART) is the most preferred therapeutic option for infertility; however, the inefficacy of presently practiced morphological evaluation criteria for embryo selection^1^ have been implicated as one of the major limiting factors that has contributed to poor pregnancy rates^2^. Given the present scenario, there is an urgent need for a reliable method that can predict the implantation potential of early embryos. The metabolic signature of the spent embryo culture medium has been proposed to be an excellent method to authenticate the viability and developmental potential of an embryo^3–5^. However, despite numerous efforts, studies have failed to arrive at a clear consensus on biomarkers that can describe an embryo with high implantation potential. This is mainly due to the variation in study designs, and patient related confounding factors. Thus, we hypothesised that an animal model would be of benefit in addressing the shortcomings of earlier reports, and would help us map changes in the metabolome in response to specific stimuli while authenticating these changes with invasive analysis. The study is aimed at charting metabolic alterations in response to induced DNA lesions at early stages of embryonic development, and using this information to demarcate embryos that are unable to undergo attachment *in vitro*.

Preimplantation stage embryos are sensitive to exogenously induced DNA lesions such as radiation^6^ and chemotherapeutic agents^7,8^ and this could be due to a peculiarity in the damage responses of the early-stage embryos^9–11^. Due to species specific and embryonic stage specific differences, cell cycle regulations vary between somatic and embryonic cells within a species. Drosophila and *Xenopus* embryos carrying DNA lesions failed to arrest, even when DNA synthesis was inhibited by aphidicolin^12,13^. Similarly, about half of the human embryos derived *in vitro* are known to possess chromosomal abnormalities, even while being developmentally and morphologically indistinguishable from euploid embryos^14,15^. This is mainly due to the fact that human embryos in the preimplantation stage are prone to genomic errors thus acquire increased incidence of DNA abnormalities which are further propagated by the increased expression of cell cycle drivers and inadequately expressed cell cycle check point regulators^16^. However, it is important to note that early cleavage embryos in primates have poorer DNA lesion repair capacity than in mice^17^ which increases the risk of abnormal reproductive outcomes when such embryos are mistaken to be healthy due to their normal morphology, and are transferred during fertility procedures. It has been shown that DNA lesions in embryos can significantly impair their implantation and post-implantation developmental competence^18^.

Transferring of embryos into pseudopregnant mice recipients is among the most commonly practiced techniques to determine their embryo viability, implantation potential, and post implantation development. However, this technique is cumbersome, time consuming, requires fine surgical skill, and limits the number of embryos that can be screened for implantation potential. As blastocysts can adhere to the surface of the petri dishes, and attached cells have the ability to proliferate *in vitro*, the inner cell mass (ICM) outgrowth assay has been suggested to be an alternative screening test to elucidate the functional competence of embryos beyond the blastocyst stage^19,20^. Moreover, a non-invasive *in vitro* assay would be useful considering the advantage of being able to monitor the proliferative ability of a large number of individual embryos at regular intervals.

In a similar retrospective approach, we were earlier able to demonstrate that biomarkers from 84 hours post injection of hCG (hpi-hCG) spent embryo culture medium could be used to predict the genetic integrity and blastocyst development 24 h in advance, using Nuclear Magnetic Resonance (NMR) spectroscopy^8^. However, these observations were limited to the developmental competence and genetic integrity of embryos until the blastocyst stage. As ICM formation serves as an *in vitro* marker to reveal the functional ability of embryos beyond blastocyst stage^21^, this study investigated the implications of metabolic profiling of 84 hpi-hCG spent embryo culture medium in predicting functional competence of peri-implantation stage embryos, using *in vitro* adherence and ICM proliferation ability.

## Results

### Embryo attachment potential is unaffected in IDL embryos

Based on previous results^8^, cis-Diamineplatinum(II) dichloride (Cisplatin, CDDP) was used to induce DNA lesions in 2-cell stage mouse embryos. At the 2-cell stage, embryos were randomly assorted to control (N = 392) and exposed to 3 µM CDDP which constituted the IDL group (N = 675). The induction of DNA lesions at the 2-cell stage resulted in a significant reduction in blastocyst formation (Control 64.5%, N = 253/392 vs IDL 48%, N = 325/675, p < 0.001) and hatching rate (Control 16.58%, N = 65/392 vs IDL 8.15%, N = 55/675, p < 0.001) on 108 hpi-hCG, even though the blastocysts from IDL group did not show any signs of morphological anomalies such as fragmentation and granulation (Table S1). Hatched blastocysts are known to attain adhesive capacity by 24 h of *in vitro* culture^22^. Attachment potential to the gelatin coated plates showed a moderate difference between the control and the IDL group (p < 0.05) after 24 h of extended *in vitro* culture (hereafter referred to as Ex 24) (control 52.65% N = 109/207 vs IDL 63.42%, N = 137/216). It is worth noting that all embryos that hatched were able to attach in both groups.

### IDL impaired ICM expansion at 96 h of extended *in vitro* culture (Ex 96)

**Figure 1:**
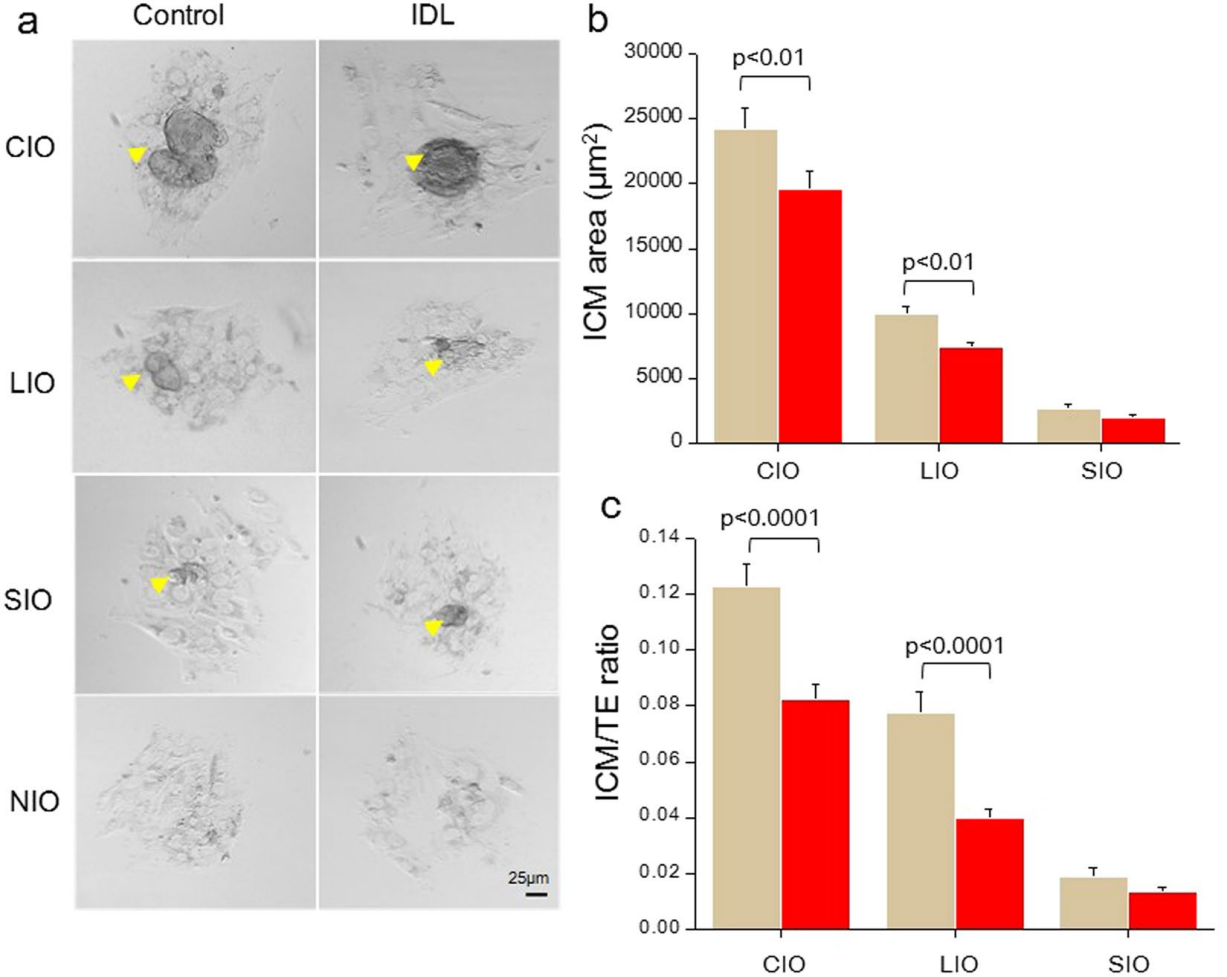
(**a**) Representative phase contrast images of inner cell mass (ICM) outgrowths on Ex 96 in control (left panel) and IDL group (right panel). Arrow head marks ICM outgrowths (Scale bar 25 µm). (**b**) Quantification of ICM area on Ex 96. ICM area was significantly reduced (p < 0.01) in Completely developed ICM Outgrowth (CIO) and Large ICM Outgrowth (LIO) derived from IDL embryos in relation to corresponding control group (**c**) ICM to trophectoderm (TE) area ratio on Ex 96 was significantly reduced (p < 0.0001) in CIO and LIO derived from IDL embryos compared to control.

ICM outgrowths (IO) were graded morphologically as completely developed ICM outgrowth (CIO), large ICM Outgrowth (LIO), small ICM outgrowth (SIO) and no ICM outgrowth (NIO), as described earlier^19^ (Fig. 1a). ICM outgrowth rate after 96 hours of extended culture (hereafter referred to as Ex 96) was marginally reduced in the IDL group, but was not statistically significant when compared to the control (control 66.18%; N = 137/207) and IDL 62.5% (N = 135/216). A significant reduction (p < 0.01) in the percentage of blastocysts that formed CIO in the IDL group was observed. Interestingly, the percentage of blastocysts that attached but failed to develop IO; i.e. the embryos with NIO, were significantly higher in the IDL group (p < 0.05, Table 1). Further, quantification of the area of outgrowth revealed that the ICM area was significantly lower in CIO (p < 0.01) and LIO (p < 0.01) of IDL group, compared to control (Fig. 1b). The ICM:TE ratio was also significantly lower in CIO (p < 0.0001) and LIO (p < 0.0001) of IDL group (Fig. 1c).

**Table 1:** Potential of IDL embryos to develop ICM outgrowths on Ex 96.

ICM grades	Completely developed outgrowth (CIO)	Large outgrowth (LIO)	Small outgrowth (SIO)	No outgrowth (NIO)
Control (N)	36.7% (76/207)	17.87% (37/207)	11.5% (24/207)	10.62% (22/207)
IDL (N)	25%* (54/216)	23.14% (50/216)	14.35% (31/216)	18.05%* (39/216)

*p < 0.05, vs control.

### CIO derived from embryos with IDL possess genetically healthy cells

**Figure 2:**
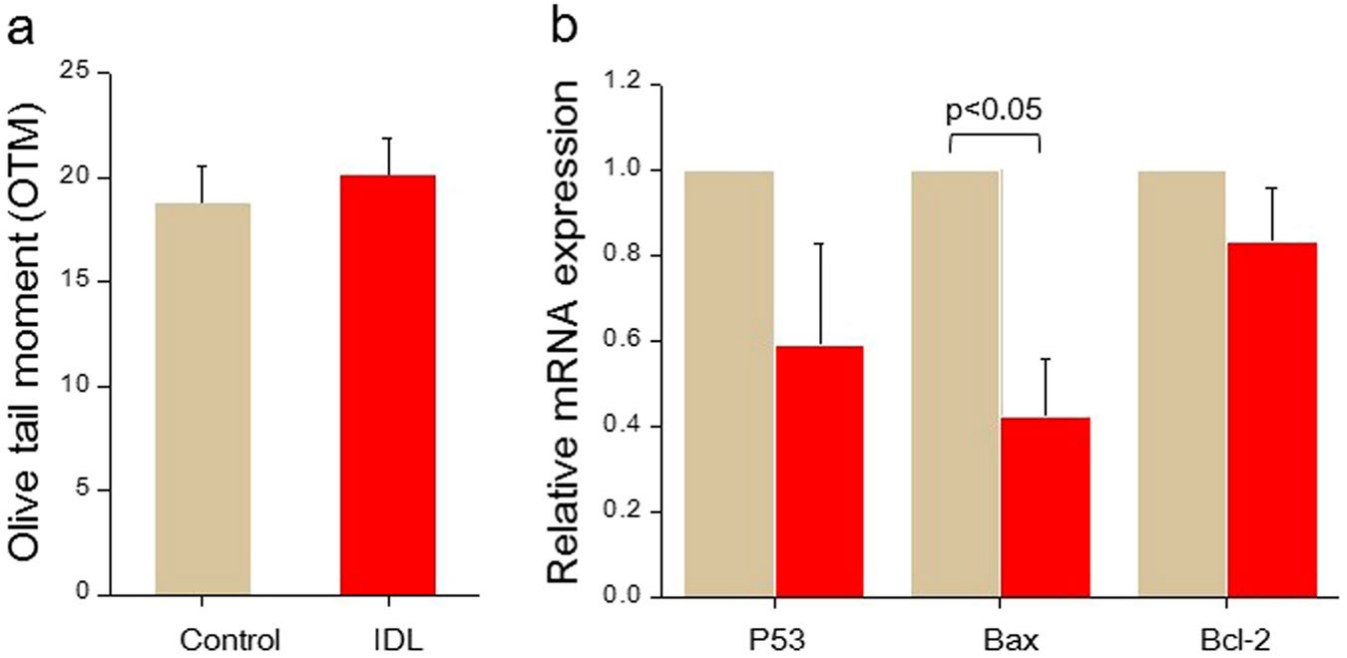
(**a**) Olive tail moment (OTM). Control (N = 13) and IDL group (N = 13) in Ex 96 outgrowths. (**b**) Relative mRNA expression of regulators of apoptosis. Expression of Bax was significantly reduced (p < 0.05) in IDL group. Light brown bars represent the control and red bars represent the IDL group. Data is represented as Mean ± SEM.

To determine the genetic integrity in CIO, the ICM cells were dissociated and assessed for the presence of DNA strand breaks using single cell gel electrophoresis (Comet assay) under alkaline conditions. Comet assay is a sensitive and quantitative tool to measure the DNA fragmentation, where damaged cells attain the shape of a comet, with the tail region consisting of fragmented DNA and the head region intact DNA. The DNA integrity in the CIO cells on Ex 96 did not vary significantly between IDL and control group (Fig. 2a). Analysis of P53 transcripts in CIO cells did not differ significantly either (Fig. 2b). Moreover, the Bax: Bcl2 ratio was 0.495, indicating the repression of apoptosis in the IDL group^23^. This observation indicates that DNA lesions induced at the 2-cell stage did not persist in the ICM population on Ex 96.

### Metabolites level on 84 hpi-hCG are related to the embryo attachment potential

**Figure 3:**
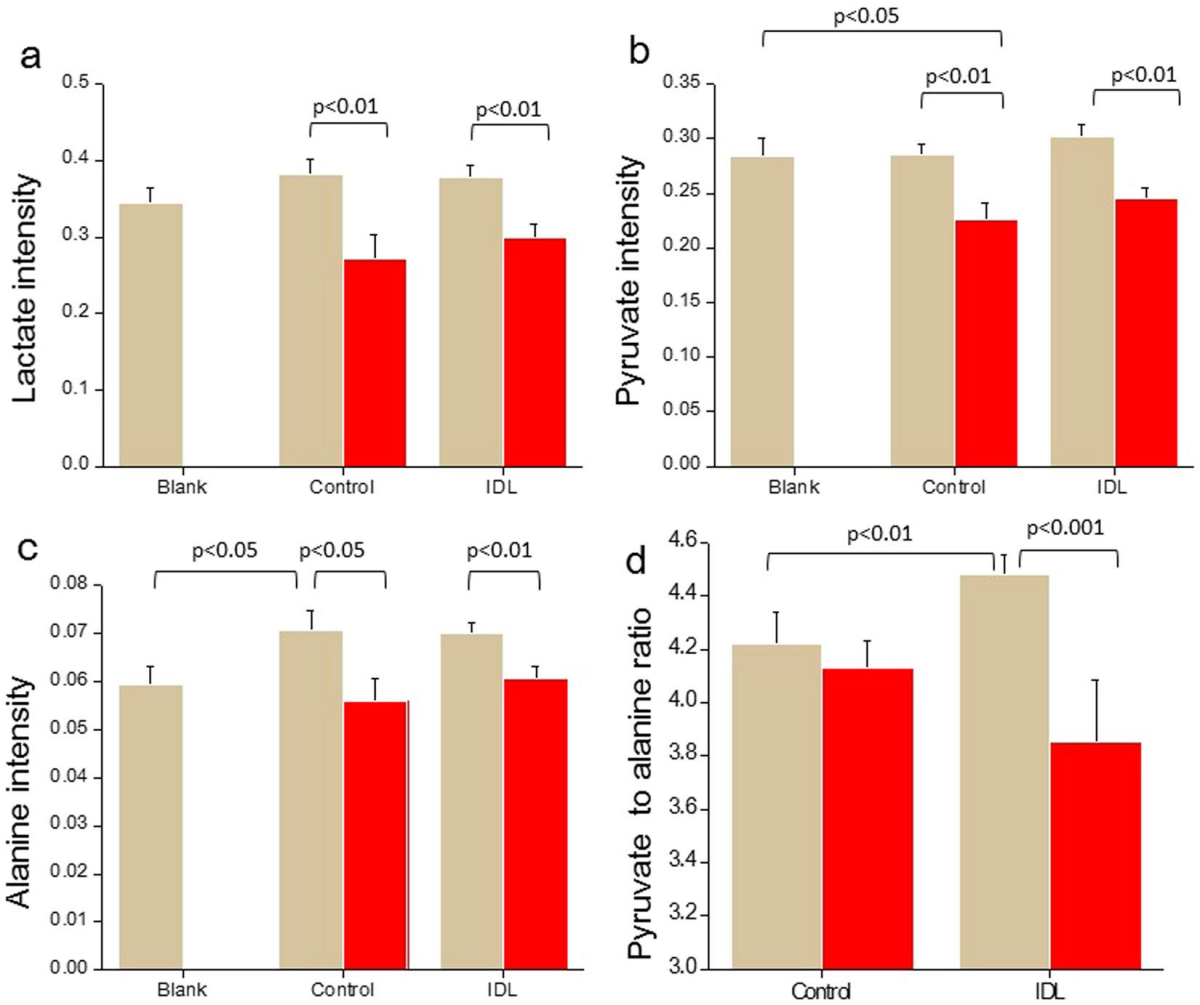
Uptake of metabolites ((**a**) lactate; (**b**) pyruvate; (**c**) alanine; (**d**) pyruvate to alanine ratio) in 84 hpi-hCG spent media of embryos in relation to attachment potential on Ex 24. In total, control N = 54, IDL N = 52 and medium control N = 12 84 hpi-hCG spent media samples were analyzed based on their attachment potential on Ex 24. Light brown bars represent attached (Control N = 33, IDL N = 33) and red bars represent unattached embryos (Control N = 21, IDL N = 19). Data represented as Mean ± SEM.

Early detection of changes in metabolic pattern has the potential to provide an immense advantage in aiding the selection of a competent embryo with high implantation potential^8,23–25^. Intensities of metabolites in the spent medium was interpreted as uptake or release, compared to the level of metabolites in culture medium without embryos which was used to determine the baseline of metabolite levels^26^. Hence, the metabolic signatures of 84 hpi-hCG spent embryo culture medium (control N = 54, IDL N = 52, medium control N = 12) was assessed noninvasively by NMR spectroscopy. The intensities of metabolites on 84 hpi-hCG were segregated based on the *in vitro* attachment potential of blastocysts on Ex 24. Embryos that failed to attach had significantly lower intensity of lactate (p < 0.01, Fig. 3a) and pyruvate (p < 0.01, Fig. 3b) in the spent medium when compared to those that attached in corresponding groups. The intensities of other metabolites which did not vary significantly across the groups are shown in Table S2. The glucose to lactate ratio was analyzed as it can serve as an indicator of glycolysis. However, no significant perturbation in the ratio was observed between attached embryos in the control and IDL group (Fig. S1). Additionally, Alanine intensity in 84 hpi-hCG spent media of embryos from control group (that later attached) was significantly higher than the medium control (p < 0.05), suggesting that these embryos released alanine into the medium (Fig. 3c). Further, alanine intensity in unattached embryos was significantly lower in both control (p < 0.05), and IDL group (p < 0.01), suggesting that embryos which failed to attach on Ex 24 utilised alanine from the culture medium.

Proline uptake was significantly higher in embryos that successfully attached (control p < 0.05 and IDL p < 0.001) than in those that failed to attach. Furthermore, the pyruvate to alanine ratio was significantly lower (p < 0.001) in IDL embryos which failed to attach (Fig. 3d). The intensities of other metabolites tested are provided in the Table S3.

Notably, though the attachment potential was identical in control and IDL embryos, not all these embryos formed CIO on Ex 96 (Table 1). Thus, the metabolic profile on 84 hpi-hCG was further categorised in relation to the ICM outgrowth potential. However, the metabolic signature on 84 hpi-hCG did not differ between embryos that grew into CIO, and those that failed to develop IO after initial attachment.

### IDL in embryos did not affect Ex 96 spent medium metabolic signature

Though comet data in ICM cells from CIO suggested the disappearance of DNA lesions by Ex 96, we investigated the possibility of delayed effects of IDL on the metabolic flux. The spent culture medium on Ex 96 (control N = 40, IDL N = 36) was subjected to NMR spectroscopy. Metabolite levels were compared between spent media of CIO of control and IDL group on Ex 96. While the glucose uptake on Ex 96 was significantly higher (p < 0.001) in CIO of IDL embryos when compared to CIO in the control group (Table 2), no significant variation in the uptake of amino acids or lactate/pyruvate was observed between embryos that developed CIO and NIO in the control and IDL groups.

**Table 2:** Relative intensities of metabolites (normalized with respect to TSP) in spent culture medium on Ex 96 in relation to ICM developmental potential on Ex 96.

	Control		IDL	
	CIO N = 26	NIO N = 14	CIO N = 23	NIO N = 13
Glucose	3.96 ± 0.10	3.96 ± 0.19	2.88 ± 0.21**	3.43 ± 0.28
Lactate	4.57 ± 0.06	4.63 ± 0.05	4.66 ± 0.11	4.65 ± 0.20
Phenylalanine	0.37 ± 0.01	0.36 ± 0.01	0.35 ± 0.02	0.33 ± 0.02
Tyrosine	0.25 ± 0.01	0.24 ± 0.01	0.26 ± 0.05	0.22 ± 0.01
Threonine	0.28 ± 0.01	0.28 ± 0.01	0.24 ± 0.01	0.25 ± 0.01
Lysine	0.75 ± 0.01	0.73 ± 0.02	0.71 ± 0.02	0.74 ± 0.02
Methionine	0.14 ± 0.01	0.14 ± 0.01	0.12 ± 0.01	0.11 ± 0.01
Glutamine	2.59 ± 0.04	2.59 ± 0.05	2.43 ± 0.06	2.43 ± 0.08
Alanine	0.47 ± 0.01	0.47 ± 0.01	0.48 ± 0.01	0.48 ± 0.02
Valine	0.84 ± 0.02	0.82 ± 0.02	0.81 ± 0.02	0.80 ± 0.03
Isoleucine	0.80 ± 0.02	0.79 ± 0.02	0.76 ± 0.02	0.76 ± 0.03

**p < 0.001 vs control.

## Discussion

This study demonstrates the correlation between metabolites levels in 84 hpi-hCG spent medium and the attachment ability of blastocysts. Pyruvate, lactate, and alanine levels in the spent media showed a significant variation between attached and unattached embryos on Ex 24, irrespective of whether the embryo was subjected to IDL or not. We propose that the aforementioned metabolites be further explored using appropriate experimental models, to prove their potential as biomarkers in the prediction of implantation ability of *in vitro* derived human embryos

### DNA lesions impede preimplantation development, but not blastocyst attachment ability and ICM genetic integrity

The induction of CDDP mediated DNA lesions at the 2-cell stage affected blastocyst formation and hatching potential. These results are in agreement with our previous observation, where embryos carrying induced DNA lesions (IDL) developed normally for the first 2.5 days (60 hpi-hCG), but began to exhibit a developmental delay at 84 hpi-hCG, though they were morphologically indistinguishable from control embryos^8^. On the other hand, the potential of hatched blastocysts to attach on Ex 24 and develop into ICM outgrowths was similar in both control and IDL groups. The IDL embryos on 108 hpi-hCG were found to be morphologically indistinguishable from the control, which points at assessing the embryos for nuclear integrity at later stages of development. It has been shown that genetic instability can persist in F1 offspring derived from DNA fragmented sperm, despite normal embryo morphology at preimplantation stage^11,18^.

During early embryonic development, the cell cycle progression is rapid and DNA replication occurs at an accelerated rate, as evidenced by short cell cycle durations^27^. Preimplantation embryos in response to genetic insult, exhibit hierarchy in checkpoint system^11,28^. The integrity of the genome is thus constantly at risk due to the errors intrinsic to DNA replication^29^. Embryos attain efficient DNA damage response pathways after implantation, and this is crucial for the proper development of the organism during this period^30–32^. In mammals, the trophectoderm (TE) forms tissues that make up the placenta, while the ICM cells proliferate rapidly to give rise to the embryo proper. Hence, the maintenance of genomic integrity within these cells is critical. A good correlation has been established between post implantation growth retardation, and impaired *in vitro* ICM outgrowth in corresponding embryos^33,34^. Though, blastocyst attachment ability was unaffected, a significant reduction in the ICM area as well as the ICM/TE ratio was observed in IDL embryos. Interestingly, comet analysis of ICM outgrowth showed intact DNA in IDL group. Further, the ICM cells in the IDL group also had a lower expression of P53, Bax, and a low Bax/BCl2 ratio, indicating an internal environment favoring repression of apoptosis. These observations suggest that ICM population in IDL embryos could eliminate or repair DNA lesions by Ex 96. However, *in vivo* studies have shown that excessive apoptosis in the ICM compartment of 84 hpi-hCG blastocysts may lead to post implantation demise, possibly due to an inadequate number of cells available to form cell lineages^11,18^. Hence, we were driven to study the metabolic behavior of cells derived from the preimplantation stage after exposure to genetic insult, which could help in identifying competent embryos.

### Modulation of embryonic metabolism by preimplantation embryos is associated with attachment potential

Genotoxic stress is known to induce alterations in metabolism, which are reflected in the surrounding culture medium. Thus, metabolomic studies on spent culture medium are extensively used for biomarker discovery that can aid in the diagnosis of pathologies and in response to toxicity^35^. Our group has demonstrated that metabolic profiling on 84 hpi-hCG is a potent indicator of apoptosis at the blastocyst stage^8^. Further, the level of spent medium metabolites on 84 hpi-hCG revealed a significant association between pyruvate, lactate, glucose, proline, lysine, alanine, valine, isoleucine and thymine, and the extent of genetic instability observed in the embryos on 108 hpi-hCG. The results from *in vitro* attachment ability extends the validity of our earlier findings^8^ in using pyruvate, lactate, alanine and pyruvate to alanine ratio as l biomarkers of implantation potential.

Here we report the excess utilisation of pyruvate on 84 hpi-hCG as a marker for impaired implantation potential in mouse embryos. Embryos exhibiting such behavior were shown to have greater than 12% apoptosis and lower cell number at the blastocyst stage^8^, and they failed to attach when subjected to ICM outgrowth assay. Pyruvate is known to act as a potent antioxidant during preimplantation development^36^. Observations in this study contradict findings in human embryos that report enhanced pyruvate uptake as biomarkers for blastocyst development and implantation potential^3,37^. However, it is worth noting that the human studies evaluated spent medium at cleavage stage (E3) while in mouse, 84 hpi-hCG corresponded to blastocyst stage where metabolic shift was observed^38^.

The utilisation of glucose by the preimplantation stage embryos is correlated with ICM outgrowth^20^. In contrast, the level of glucose or glucose to lactate ratio in the present study did not differ significantly between control and IDL embryos that attached. However, a significantly increased (p < 0.01) glucose to lactate ratio on 84 hpi-hCG was found in IDL embryos that failed to undergo attachment, implying a higher rate of glycolysis often associated with metabolically active embryos carrying DNA lesions as reported earlier by our group^8^.

Low pyruvate to alanine ratio on 84 hpi-hCG observed in this study is indicative of the failure to undergo *in vitro* attachment (Fig. 3d). The conversion of pyruvate to alanine is an established mechanism to combat excess ammonium that is generated due to the spontaneous breakdown of nutrients during extended cultures^39^. This observation is an indirect indicator of an oxidatively stressed internal environment and loss in embryonic viability. Additionally, embryos that failed to attach also had increased lactate consumption. Lactate can be converted to pyruvate, and can aid in the generation of ATP via the TCA cycle. Excess requirement of energy substrates is common in embryos with increased DNA damage as the DNA repair is an energy expensive process^40–42^.

Amino acids are crucial in ensuring optimal development in preimplantation embryos^43^. Proline uptake was higher in embryos that attached and formed well-developed outgrowths in both groups when compared to embryos that failed to attach. Proline is known to play a key role in inducing differentiation of embryonic stem cells^44^ and improving preimplantation development^45^. Moreover, proline can be converted to glutamate, which participates in the transamination reaction catalysed by alanine transaminase. However, the proline level in the present study is opposite to pyruvate and lactate levels and the reason behind this trend cannot be currently explained.

It has been shown that TE and ICM have distinct energy requirements and metabolic pathways, suited for their respective functions^46,47^. Due to their high proliferative nature, ICM cells use aerobic glycolysis for increasing biomass^48,49^. Analysis of Ex 96 spent medium did not show any difference in the level of metabolites between control and IDL groups, except for increased glucose uptake in the IDL group. It has been shown that mitochondria in the ICM have a lower membrane potential^50^, which is indicative of reduced mitochondrial function.

Our data suggests that metabolic profiling of spent embryo culture medium on 84 hpi-hCG could be a valuable adjunct to morphological parameters, in determining embryonic viability and attachment potential of preimplantation embryos; thus aiding the selection of a viable embryo with high implantation potential. It is interesting to note that ICM outgrowths derived from embryos subjected to genotoxic stress had intact DNA, despite having increased TUNEL positive cells on 108 hpi-hCG. Further studies are required to address the mechanism behind the phenomenon. One of the major limitations of this study is that we have assessed only the DNA integrity and not evaluated incidences of mutations, aneuploidy, or epigenetic alterations in the well-developed ICM cells. In a similar experimental set up, our earlier study has shown an almost two-fold increase in the DNA damage at 108 hpi-hCG^8^. However, it is important to note that the possibility of DNA intact ICM population having originated from unaffected 2-cell stage embryo cannot be ruled out. With respect to DNA integrity, embryos that developed to healthy outgrowths did not exhibit any changes in metabolism. Differences between groups in metabolic measurements are too small to be considered useful as biomarkers in a clinical setup. In addition, our study looked into only the association between metabolites and *in vitro* attachment and ICM proliferative ability, but not the *in vivo* implantation potential which we believe to be the most appropriate functional marker. Further studies in this direction can provide useful insight on the interaction between metabolic and molecular signaling pathways involved in embryonic survival. Careful attention must be given to the different developmental timelines between human and mouse embryos while extrapolating these findings to the clinical set up.

## Methods

All experiments and animal handling were conducted in accordance with the institutional guidelines for animal experimentation after obtaining prior approval from Kasturba Medical College Institutional Animal Ethics Committee (IAEC/KMC/24/2012). Eight-week old healthy Swiss albino male and female mice, maintained under controlled conditions of temperature (23 ± 2 °C), light (12 h light/dark cycles), standard diet, and water *ad libitum* were used in this study. Embryos at the 2-cell stage were retrieved from successfully mated female mice primed with 5 IU pregnant mare serum gonadotropin (PMSG, Cat No. G4877, Sigma Aldrich, USA) and 10 IU human chorionic gonadotropin (hCG, Ovitrig, administered 48 h after PMSG). Embryos retrieved at 36 h post hCG were washed and cultured in droplets of pre-equilibrated 30 µL Innovative Sequential Medium (ISM1, Cat No. 10500010, Origio, Denmark) at 37 °C and 5% CO_2_ under oil. Please note that embryos are cultured singly to enable the analysis of metabolites related to the individual embryo. Embryos remain in the same droplets for a period of 48 h (84 hpi-hCG) and replaced by the same medium for an additional culture period of 24 h until 108 hpi-hCG.

### Induction of DNA lesions in embryos

**Figure 4:**
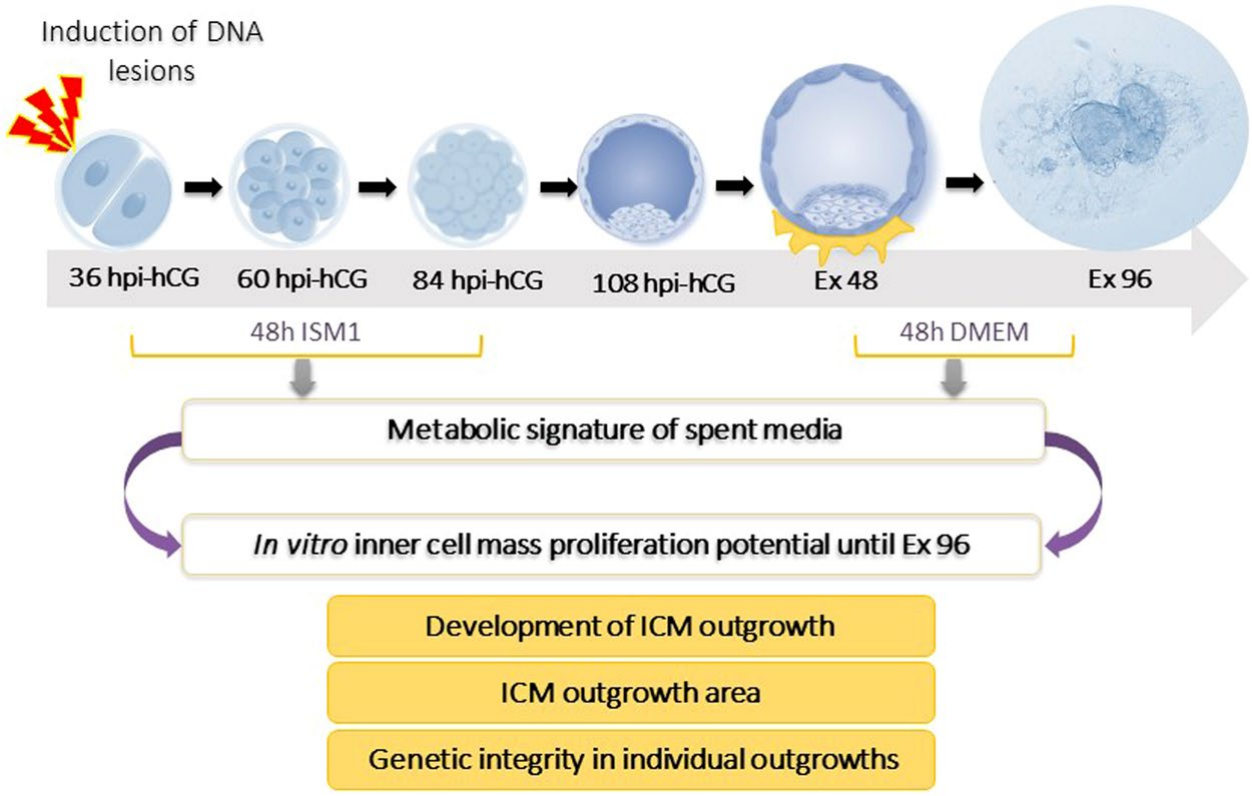
Overview of the experimental design.

A schematic representation of the methodology employed in this study is illustrated in Fig. 4. Antineoplastic agent cis-Diamineplatinum(II) dichloride, (Cisplatin (CDDP), Cat No. P4394, Sigma Aldrich, USA) was used to induce DNA lesions (IDL) in embryos at the 2-cell stage. The concentration and duration of CDDP exposure was determined based on preliminary experiments conducted to identify a treatment that would induce adequate DNA damage while also allowing embryo development to the blastocyst stage^8^ (Table S4). Following exposure to 3 µM CDDP for 2 h, the embryos were washed 3 times in ISM1 medium to remove all traces of the drug, and cultured individually in droplets of 30 µL ISM1 at 37 °C and 5% CO_2_ under oil. For every six embryos, one medium droplet without an embryo was maintained, which served as an internal control (henceforth referred to as medium control). The embryos were evaluated at 24 h intervals under a phase contrast inverted microscope (Olympus IX-71, Japan). After 48 h of culture on 84 hpi-hCG, the embryos were transferred to fresh droplets of ISM1 medium and cultured until 108 hpi-hCG. The spent embryo culture medium on 84 hpi-hCG was collected after thorough mixing and individually placed into labelled 0.5 mL tubes, paraffin sealed, snap frozen in liquid nitrogen, and stored at -80 °C until analysis.

### ICM outgrowth assay

Blastocysts appearing morphologically normal with expanded blastocoel on 108 hpi-hCG were selected for ICM outgrowth assay, which was performed as described as earlier^19^. Briefly, multi well dishes (Sigma, Cat No. D7039) were precoated with 0.1% gelatin (Sigma-Aldrich, Cat No. G1393) for 30 min at room temperature. Excess gelatin was removed and the dishes were air dried. Individual blastocysts on 108 hpi-hCG were transferred into each well containing 500 µL DMEM (Sigma-Aldrich, Cat No. D5648) supplemented with 20% fetal calf serum (FCS, Sigma-Aldrich, Cat No. F4135) and cultured until Ex 96. The culture medium was changed after 48 h of extended *in vitro* culture (Ex 48). Blastocyst attachment and proliferation were monitored at 24 h intervals under an inverted phase contrast microscope (IX 73, Olympus, Japan). The spent culture medium was snap frozen on Ex 96 for metabolic profiling. The ICM and TE area were quantified using ProgRes CapturePro (Version 2.7.7, Jenoptik, Germany) by outlining the boundaries of the ICM and TE (Fig. S2).

### RNA extraction and quantitative real-time PCR (qPCR)

Total RNA was extracted from 15 completely developed ICM outgrowths using RNAqueous micro kit (Cat No. AM1931, Ambion, Life Technologies, USA) according to the manufacturer’s instructions followed by treatment with DNAse 1. cDNA was synthesized using Protoscript First strand cDNA synthesis kit (Cat No. E6300S, New England Biolabs Inc, USA). 1 µL cDNA was used to determine the relative mRNA levels of P53, Bax and Bcl-2 with SYBR Green detection (Cat No. RR420, Takara, Japan) on a Step One Real time PCR system (Applied Biosystems, USA). Primer sequences for qRT-PCR are provided in supplementary information (Table S5). Exon-spanning primers were used to avoid amplification of genomic DNA. The transcript levels were normalized to GAPDH. The amplification conditions for all primer pair were as follows: 20 s incubation at 95 °C, amplification at 95 °C for 1 s and annealing at 60 °C for 20 s for 40 cycles. During the extension step of each cycle, the fluorescence emitted at each cycle was collected for an entire period of 30 s. qPCR data was analysed as described before.^8^ Briefly, melt curve was used to assess the homogeneity of the PCR amplicon. Mean Ct values generated in the samples were computed and normalized to GAPDH. The 2^-ΔΔCt^ method was used to determine the relative expression levels in terms of fold change^51^. Reactions were performed in triplicates in a total volume of 20 µL, and a minimum of 3 biological and 3 technical replicates were assessed.

### Assessment of DNA fragmentation in ICM outgrowth cells by Alkaline Comet Assay

DNA damage in completely developed ICM outgrowths was assessed by Comet assay as described earlier^52^ with minor modifications. Briefly, the outgrowths were washed in phosphate buffered saline (PBS) to remove the culture medium. TE cells tightly adhere to the base of the dish while ICM outgrowths project upwards (Fig. S5). Thus, ICM aggregates from completely developed outgrowths were dislodged by gentle pipetting in a cell dissociation buffer (Ca^2+^-Mg^2+^ free DPBS containing 0.8% EDTA). Individual ICM aggregates were extracted and incubated in a dissociation buffer for 8 min. The outgrowth was dissociated in 10 μL of 0.05% Trypsin maintained at 37 °C for 1 min, followed by disaggregation into a single cell suspension by gentle pipetting. The reaction was terminated using 10 μL DMEM with 0.5% FCS.

The single cell suspension was loaded onto equal volume of low melting point agarose (LMPA, SRL, Cat No. 0140151.) on a slide precoated with 1% normal melting point agarose (NMPA, SRL, Cat No. 0144162) and allowed to solidify on an ice pack followed by the addition of 100 μL of 0.8% LMPA. The slide was immediately placed in ice cold lysis solution (2.5 M NaCl, 100 mM EDTA, 10 mM Tris, 1% Triton X-100 and 10% DMSO, pH 10.0) and incubated at 4 °C overnight. The DNA unwinding was carried out by immersing the slides in an electrophoresis buffer (300 mM NaOH and 1 mM EDTA, pH 13.0) for 25 min followed by electrophoresis at 12 Volts for 10 min (pH < 13). The slide was neutralized with 0.4 M Tris-HCl (pH 7.4) for 10 min and fixed in chilled absolute ethanol. For visualization and scoring, the slides were rehydrated in cold PBS for 10 minutes and stained with 2 µg/ml ethidium bromide. The slides were observed under a fluorescent microscope (Imager-A1, Zeiss, Germany) and images were captured using the 40 X objective. A minimum of 50 randomly selected images were captured from each slide. The cell with DNA damage attains the shape of a comet with the tail region consisting of fragmented DNA and the head region with intact DNA (Fig. S3). The comet evaluation of the captured images was done using Kinetic Imaging software (Komet 5.5, UK). Excessively large cells representing trophectoderm contamination were eliminated from analysis. The amount of DNA damage in each cell, as determined by comet assay, is represented as Olive Tail Moment (OTM), which is defined as the product of the length of the comet tail and the content of fragmented DNA in the tail region^53^.

### Metabolic profiling by NMR spectroscopy

The metabolic profile of spent media samples collected from blastocysts that were used to ICM outgrowth assay was carried out on a Bruker AVANCE NMR spectrometer operating at a ^1^H resonance frequency of 800 MHz equipped with a cryogenically cooled triple resonance (^1^H, ^13^C, ^15^N) probe. All spectra were acquired at 298 K.

#### NMR experiments for metabolic profiling of ISM1

The spent ISM1 samples were thawed at room temperature 30 min prior to sample preparation. After vortexing, 25 µL of ISM1 spent media was diluted to 550 µL of a solution containing pre-added sodium salt of 2,2,3,3 tetradeutero 3-(trimethyl silyl) propionate (TSP, Sigma, Cat No. 269913) in ^2^H_2_O. TSP was used as a standard reference molecule. All data were analysed using the Bruker TOPSPIN 3.5 software. All peak integrals were measured with respect to the corresponding integral/area of the TSP signal (which was normalized to 1.0). The signals of metabolites from the medium were assigned as described previously^24,54^.

Signals from the protein component of the media were suppressed using a Carr-Purcell-Meiboom-Gill (CPMG) sequence of 20 ms delay, which was incorporated into the one-dimensional (1D) radio-frequency pulse scheme^24^. Suppression of the water (H_2_O) was achieved with pre-saturation (r.f. strength = 50 Hz). The 1D ^1^H NMR spectra was acquired using a ^1^H 900 pulse width of 8 µs, relaxation delay (trel) of 7 s between scans, spectral width of 9600 Hz and an acquisition time (tmax) of 1.1 s (16 K complex points). The total of trel + tmax~8 s was used to ensure that it exceeds 3*T1 of the metabolites being studied. A total of 400 transients were collected, resulting in a measurement time of 53 min for each sample. The time domain data was apodized with an exponential window function (line broadening 0.3 Hz) and zero-filled to 64 K points prior to Fourier transform.

#### NMR experiment for metabolic profiling of DMEM

The spent DMEM samples were thawed to room temperature 30 min prior to sample preparation. After vortexing, 50 µL of TSP in ^2^H_2_O was added to 450 µL of DMEM spent media. Parameters for data acquisition were same as that used for ISM1 samples except for 32 transients that were used to record the spectrum, resulting in a measurement time of 5 min for each sample^55^.

### Statistical Analysis

All data analysis was performed using GraphPad Prism Version 5.0 (GraphPad Software, Inc., USA). The data represented as percentage was evaluated by Chi Square statistics. For the variables expressed as Mean ± SEM (Standard error of mean), statistical significance was evaluated by unpaired t-test for normally distributed data and Mann-Whitney U-test for data that did not conform to normal distribution. p < 0.05 was considered statistically significant. The graphs were plotted using Microcal Origin 6.0 (USA).

## Electronic supplementary material

Supplementary information
