# Supplementary material for: Spent embryo culture medium metabolites are related to the in vitro attachment ability of blastocysts
Source: Sci Rep. 2018 Nov 19;8:17025. doi: 10.1038/s41598-018-35342-2 (PMC6242932; doi:10.1038/s41598-018-35342-2)
Supplement: Supplementary file 1 — Supplementary information [file 41598_2018_35342_MOESM1_ESM.pdf]

Title: Spent embryo culture medium metabolites are related to the *in vitro* attachment ability of blastocysts

Author affiliation: Fiona D'Souza[1],Shubhashree Uppangala[1,] Gitanjali Asampille[2,3], Sujith Raj Salian[1], Guruprasad Kalthur[1], Riccardo Talevi[4], Hanudatta S Atreya[2,3], Satish Kumar Adiga[1]

[1]Department of Clinical Embryology, Kasturba Medical College, Manipal Academy of Higher Education, Manipal. [2]NMR Research Centre and [3]Solid State and Structural Chemistry Unit, Indian Institute of Science, Bangalore, India. [4]Dipartimento di Biologia, Università di Napoli "Federico II", Complesso Universitario di Monte S Angelo, Napoli, Italy

Corresponding authors:

Satish Kumar Adiga, Ph.D.

Department of Clinical Embryology, Kasturba Medical College, Manipal Academy of Higher Education, Manipal-576 104, India Tel: 91-820-29-22320,

E mail: satish.adiga@manipal.edu

Hanudatta S. Atreya, Ph.D

NMR Research Centre, Indian Institute of Science, Bangalore-560012, India

Email: hsatreya@iisc.ac.in

Supplementary table 1: Preimplantation developmental pattern in IDL embryos

	36 hpi-hCG	60 hpi-hCG		84 hpi-hCG		108 hpi-hCG		
	2c	4c	6-8c	Morula	Early Blastocyst	Early Blastocyst	Expanded Blastocyst	Hatched Blastocyst
**Control**	100% (392/392)	29.08% (114/392)	65.82% (258/392)	34.44% (135/392)	42.60% (167/392)	11.73% (46/392)	36.22% (142/392)	16.58% (65/392)
**IDL**	100% (675/675)	50.52%** (341/675)	33.19%** (224/675)	43.11%* (291/675)	18.67%** (126/675)	16.14% (109/675)	23.85%** (161/675)	8.15%** (55/675)

*p<0.01, **p<0.001 vs control.

Supplementary table 2: Metabolite intensities in 84 hpi-hCG spent media of embryos in relation to attachment potential on Ex 24.

	Blank	Control		IDL	
	N=12	Attached N=33	Unattached N=21	Attached N=33	Unattached N=19
Glucose	0.040 ± 0.005	0.040 ± 0.003	0.034 ± 0.004	0.045 ± 0.003	0.044 ± 0.003
Lysine	0.068 ± 0.010	0.067 ± 0.005	0.069 ± 0.012	0.073 ± 0.005	0.065 ± 0.005
Thymine	0.105 ± 0.007	0.110 ± 0.005	0.098 ± 0.007	0.110 ± 0.005	0.105 ± 0.004
Valine	0.139 ± 0.009	0.161 ± 0.009	0.138 ± 0.014	0.157 ± 0.007	0.131 ± 0.005
Isoleucine	0.070 ± 0.007	0.073 ± 0.006	0.073 ± 0.010	0.069 ± 0.003	0.076 ± 0.012

No Significant differences between the groups

Supplementary table 3: Metabolite intensities in 84 hpi-hCG spent media of embryos in relation to hatching rate on 108 hpi-hCG development

	**Control**		**IDL**	
	Hatched N=19	Unhatched N=35	Hatched N=8	Unhatched N=44
Glucose	0.037 ± 0.005	0.037 ± 0.003	0.037 ± 0.007	0.046 ± 0.002
Lactate	0.373 ± 0.027	0.322 ± 0.024	0.343 ± 0.044	0.345 ± 0.016
Pyruvate	0.283 ± 0.016	0.261 ± 0.015	0.268 ± 0.032	0.285 ± 0.009
Alanine	0.070 ± 0.007	0.063 ± 0.003	0.057 ± 0.007	0.087 ± 0.020
Proline	0.031 ± 0.003	0.035 ± 0.003	0.037 ± 0.007	0.035 ± 0.002
Lysine	0.069 ± 0.008	0.067 ± 0.008	0.056 ± 0.014	0.073 ± 0.004
Thymine	0.103 ± 0.008	0.107 ± 0.005	0.098 ± 0.012	0.109 ± 0.004
Valine	0.153 ± 0.014	0.151 ± 0.010	0.136 ± 0.020	0.151 ± 0.005
Isoleucine	0.072 ± 0.009	0.074 ± 0.006	0.064 ± 0.009	0.077 ± 0.005

No Significant differences between the groups

Supplementary table 4: Effect of varying concentrations of CDDP exposure on blastocyst rate and TUNEL labeling index

	**Blastocyst rate (%)**	**TUNEL labeling index**
**Control (n= 46)**	63.04 (29/46)	2.6 ±0.36
**1.5 µM (n= 34)**	58.82 (20/34)	5.80± 0.64
**3 µM (n= 40)**	42.5 (17/40)	8.11±1.23*
**6 µM (n= 35)**	22.85 (8/35)**	27±3.9**

*p < 0.01, **p <0.001 vs control

Supplementary table 5. Primer sequences for qRT-PCR

Gene	Forward Primer	Reverse Primer
P53	5’-GACCGCCGTACAGAAGAAGA-3’	5’-GCGGATCTTGAGGGTGAAATA-3’
Bax	5’- ATCTGGTTCTGCAAGCGTTTA-3’	5’-CCTGCTCCGAATTTGGTGAAA-3’
Bcl-2	5’- ATGCCTTTGTGGAACTATATGGC- 3’	5’-GGTATGCACCCAGAGTGATGC-3’
GAPDH	5’- AGGTCGGTGTGAACGGATTTG- 3’	5’-TGTAGACCATGTAGTTGAGGTCA-3’

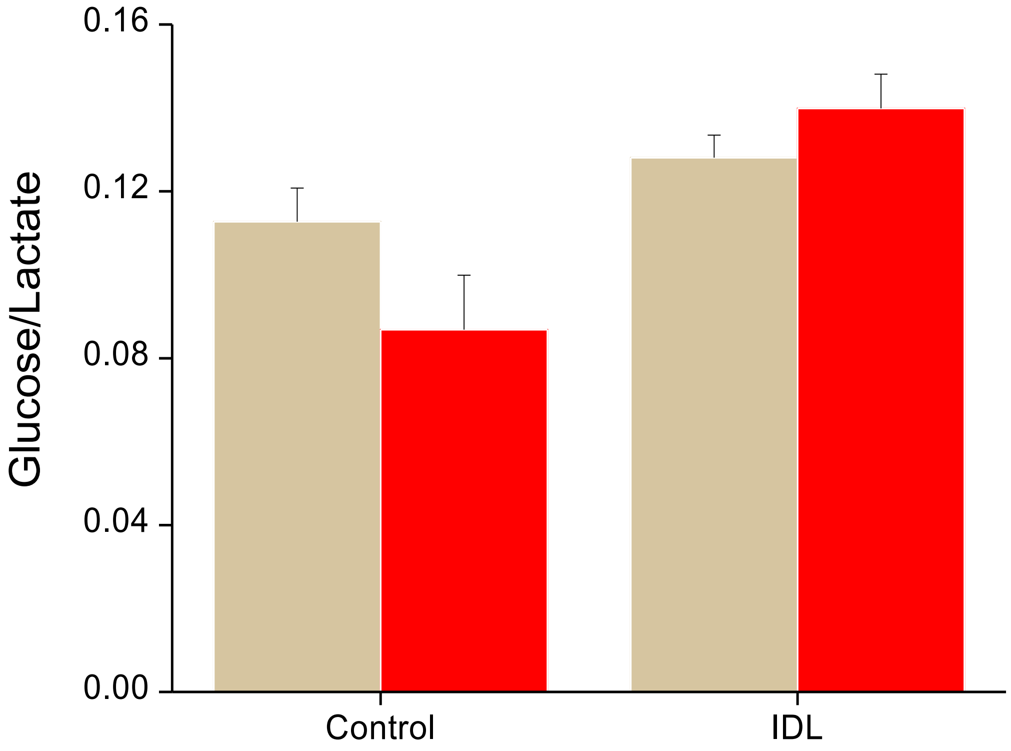

Fig S1 The glucose to lactate ratio in E 84 hpi-hCG spent media of embryos. Light brown bars represent attached and red bars represent unattached embryos. Data represented as Mean ± SEM.

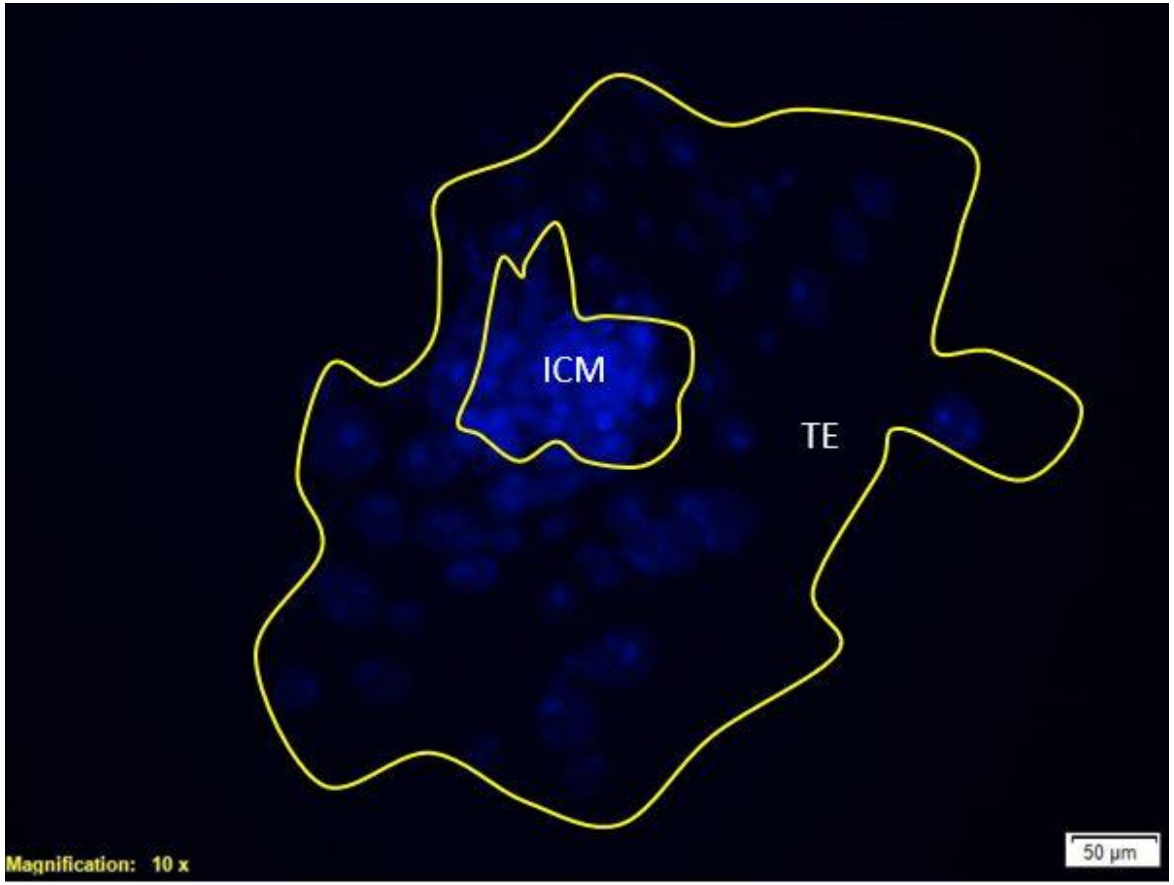

Fig S2 ICM outgrowth assay measurement: The ICM and TE area were quantified using ProgRes CapturePro (Version 2.7.7, Jenoptik, Germany) by outlining the boundaries of the ICM and TE

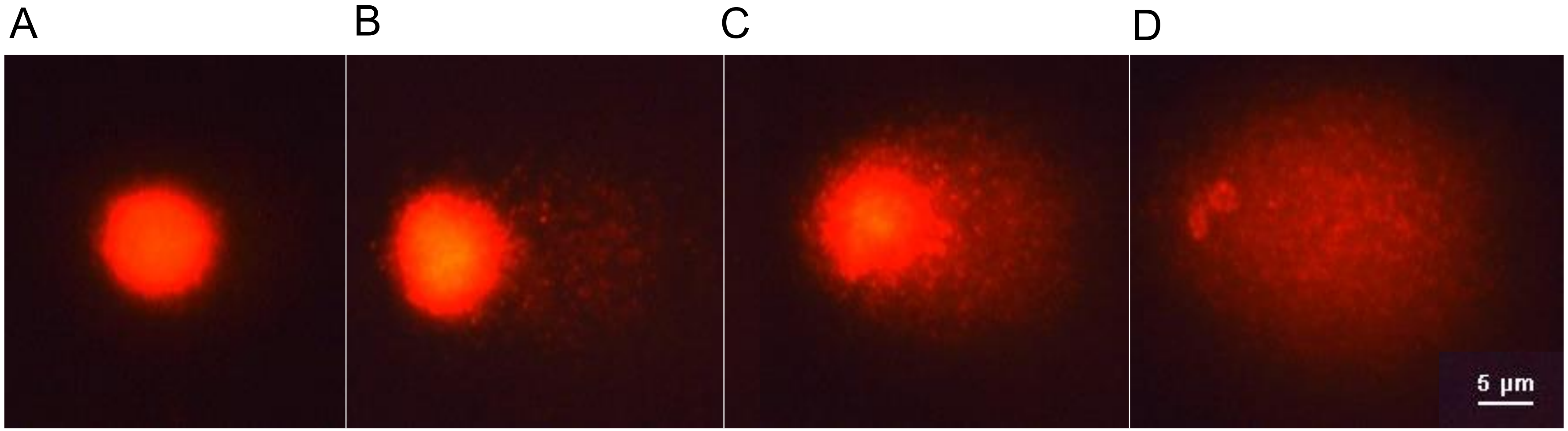

Fig S3 Comet images of spermatozoa showing varying levels of DNA damage A) Sperm head with intact DNA. B) moderate DNA damage. C) high DNA damage. D) severe DNA damage
